# Evaluation of antigout activity of *Phyllanthus emblica* fruit extracts on potassium oxonate-induced gout rat model

**DOI:** 10.14202/vetworld.2015.1230-1236

**Published:** 2015-10-23

**Authors:** Vaidehi N. Sarvaiya, Kamlesh A. Sadariya, Prakash G. Pancha, Aswin M. Thaker, Aashish C. Patel, Ankit S. Prajapati

**Affiliations:** 1 Department of Veterinary Pharmacology and Toxicology, College of Veterinary Science and Animal Husbandry, Anand Agricultural University, Anand, Gujarat, India; 2 Department of Animal Genetics and Breeding, College of Veterinary Science and Animal Husbandry, Anand Agricultural University, Anand, Gujarat, India; 3 Department of Veterinary Medicine, College of Veterinary Science and Animal Husbandry, Anand Agricultural University, Anand, Gujarat, India

**Keywords:** antigout activity, gout rat model, *Phyllanthus emblica* fruits, potassium oxonate

## Abstract

**Aim:**

The present study has been conducted to evaluate antigout activity of aqueous and alcoholic extracts of *Phyllanthus emblica* fruits following its 28 days repeated oral administration on potassium oxonate-induced gout rat model.

**Materials and Methods:**

The study was conducted on 42 male Sprague-Dawely rats dividing them in seven groups having six rats in each group. Groups I, II, and III served as vehicle control group, gout control group, and standard treatment control group, respectively. Rats of all the groups except vehicle control group were administered potassium oxonate at 250 mg/kg (IP), throughout the study period (28 days) for induction of gout. Groups IV and V received aqueous extract of *P. emblica* at 200 and 400 mg/kg, and Groups VI and VII received alcoholic extract of *P. emblica* at 200 and 400 mg/kg (daily oral for 28 days). At the end of study, all the rats were subjected to blood collection; blood and serum sample were analyzed for hematological and biochemical parameters, respectively. After collection of blood samples on the 29^th^ day, all the rats were sacrificed and subjected to post mortem examination to determine the presence or absence of gross and histopathological lesions in kidney tissues.

**Results:**

At the end of study, rats of gout control group showed increase in platelets counts, serum creatinine, uric acid, blood urea nitrogen (BUN), and xanthine oxidase (XO) enzyme level along with alterations in kidney tissues as compared to vehicle control group. Gouty rats treated with aqueous and alcoholic extracts of *P. emblica* at 200 and 400 mg/kg body weight and standard treatment allopurinol at 5 mg/kg body weight showed reduction in platelets counts, serum creatinine, uric acid, BUN, and XO enzyme level along with significant improvements in histological structure of kidney as compared to rats of gout control group.

**Conclusion:**

Oral administration of aqueous and alcoholic extracts of *P. emblica* fruits for 28 days has shown protection against gout in dose-dependent manner in rats.

## Introduction

Gout is a heterogynous group of diseases resulting from the deposition of urate (as monosodium urate monohydrate) crystals in supersaturated extracellular fluids. These crystals cause an acute inflammatory response and can induce a permanent tissue damage which is characterized by the appearance of ulceration of the joint cartilage, marginal osteophytosis, erosive lesions and chronic inflammation of synovial membrane [1]. The underlying metabolic disorder in gout is an excessive concentration of uric acid in the blood.

Urate lowering therapy is the main approach in the treatment of gout. The target level of serum uric acid is <6.8 mg/dL to dissolve the urate crystals and inhibit gout attack [2,3]. The most important approach in the treatment of gout is the development of xanthine oxidase (XO) inhibitors, which are effective in reducing plasma and urinary urate levels and reverses the development of tophaceous deposits [4]. So, food components which inhibit XO activity can reduce the formation of uric acid and alleviate inflammation. It is because of XO enzyme which has an important role in hyperuricemia, catalyzing the oxidation of hypoxanthine to xanthine and then to uric acid [5].

Allopurinol is the most common clinically used XO inhibitor prescribed for the treatment of gout [6]. Allopurinol can cause the side effects, such as nephrolithiasis, allergic reaction and increase the toxicity of 6-merkaptopurin [7]. Thus, the development of novel hypouricemic agents with greater efficacy and a broader safety profile is greatly needed.

In recent times, focus on plant research has increased all over the world, and a large number of evidence has collected to show immense potential of medicinal plants used in various traditional systems. Amla (*Phyllanthus emblica*) is extensively used as a rejuvenator in Ayurveda. *P. emblica* are widely used in the Indian system of medicine and believed to increase defense against diseases. Amla possess a vast ethno medical history and represents a phytochemical reservoir of heuristic medicinal value. It has been suggested in the scientific literature that consumption of *P. emblica* alleviates arthritic pain and gout. Many pharmacological studies have showed that this plant extracts having antioxidant, anticarcinogenic, antitumor, antigenotoxic, antigout, and anti-inflammatory activities, and supporting its traditional uses [8].

As per our knowledge, there was lack of reported references regarding antigout activity of aqueous and alcoholic extracts of *P. emblica* on gout rat model so the present study was planned to explore antigout effect of *P. emblica* fruits extracts following its oral administration at various doses along with hemato-biochemical and histopathological evaluation.

## Materials and Methods

### Ethical approval

The protocol of the experiment was approved by the Institutional Animal Ethics Committee with approval number 153/VPT/2013, College of Veterinary Science and Animal Husbandry, Anand, Gujarat and protocols were followed according to the guidelines of Committee for the Purpose of Control and Supervision of Experiments on Animals (CPCSEA), Government of India (Reg. No. 486/01/CPCSEA).

### Experimental animals

The study was conducted on 42 healthy male Sprague-Dawley rats of 8-12 weeks of age. Rats were procured from Zydus Research Centre (ZRC), Ahmedabad, India. The animals were housed in standard polypropylene cages (three rats per cage) and maintained under controlled room temperature (22±2°C) and humidity (55±5%) with 12 h light and 12 h dark cycle. All the rats were fed with commercially available rat normal pellet diet, and deionized water was provided *ad-libitum* throughout the course of the experiment. All the rats were kept under acclimatization for 5 days prior to grouping and initiation of the experiment. Rats were kept under constant observation during entire period of study. All necessary managemental procedures were adopted to keep the rats free from stress.

### Drugs and chemicals

Potassium oxonate (Oxonic acid potassium salt), allopurinol, and XO activity assay kit were purchased from Sigma-Aldrich (Spruce Street, St. Louis, MO, USA). Glycerin (Glycerol about 98% Purified) was purchased from Merck Specialties Pvt. Ltd. India. Reagents used for serum biochemical analysis were purchased from Coral Clinical System, Goa, India.

### Collection of plant materials

**Figure-1:**
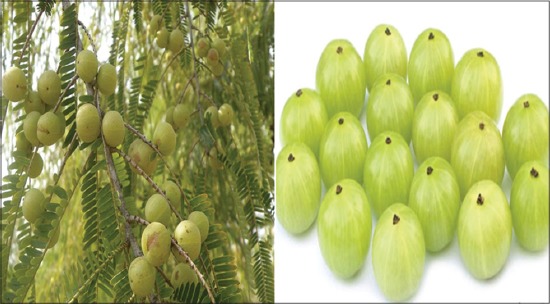
Photograph showing fruits of *Phyllanthus emblica* (Amla).

Fresh amla (*P. emblica*) fruits (Figure-1) were purchased from the Medicinal and Aromatic Plants Unit, Anand Agricultural University, Anand (Voucher No. of the specimen: AAU/MAPU/2013/386377) in December month, and they were authenticated and identified by botanist.

### Preparation of extracts

Small pieces of *P. emblica* fruits were taken and dried under shade, then powdered by mechanical grinder, sieved (sieve no: 10/44) and stored in air tight containers. The dried powder of *P. emblica* was separately placed in a continuous extraction apparatus and subjected to successive extraction using water and alcohol. Exactly, 100 g of coarse powdered material of *P. emblica* was successfully extracted in soxhlet extractor with water as well as alcohol. The extracts obtained were concentrated in rotary evaporatorat 50-60°C under reduced pressure leaving a dark brown residue. Aqueous and alcoholic extracts of *P. emblica* obtained was transferred to a petri dish and kept over water bath (50°C) until the solvent gets completely evaporated. It was stored at 4°C for further use.

### Acute toxicity testing of plant extracts

The acute oral toxicity study was carried out as per Organization for Economic Cooperation and Development (OECD) guideline No. 423. Sprague-Dawley rats were taken for the study and dosed once with 2000 mg/kg, orally. The treated rats were monitored for 24 h and up to 14 days for general clinical signs and symptoms, as well as mortality. It was observed that the aqueous and alcoholic extracts of *P. emblica* have no toxic effect on rats even at 2000 mg/kg doses, respectively. Reported study provides critical evidence for ascertaining the safety of the standardized (LD50>1000 mg/kg) that could be used as medicine along with absence of untoward effect. So, we kept our dose little lower and in two upward levels, i.e., 200 mg/kg and 400 mg/kg body weight for our study [9].

### Test compound preparation

Potassium oxonate (PO) (250 mg/kg body weight, IP) used for induction of hyperuricemia and allopurinol (5 mg/kg body weight, PO) used as a positive control were dissolved in 0.9% saline solution. Aqueous and alcoholic extracts of *P. emblica* were dispersed in water using 10% glycerin solution. For the vehicle control group, the vehicle was prepared through dissolving the same amount of glycerin in water.

### Induction of gout in rats

Experimentally-induced gout in rats (due to inhibition of uricase enzyme with PO) was used to study antigout activity of *P. emblica* fruits [10]. All the animals except vehicle control group were administered PO at 250 mg/kg body weight intraperitoneally by dissolving it in 0.9% saline solution, throughout the study period (28 days) and then after 1 h all those rats were administered test compounds.

### Experimental design

Forty two male Sprague-Dawley rats were randomly divided into seven groups (6 rats per group). Three groups served as control (one healthy control, second gout control and third positive control), where rats did not receive any extracts. Allopurinol used as a positive control, administered at 5 mg/kg of body weight orally to animals of Group III. Groups IV and V administered aqueous extracts of *P. emblica* at 200 and 400 mg/kg and Groups VI and VII administered alcoholic extracts of *P. emblica* at 200 and 400 mg/kg, respectively (daily oral for 28 days). The extracts and allopurinol solutions were administered to rats directly in stomach by using rat oral gavage needle with 2 ml BD syringe.

### Collection of blood and tissue samples

At the end of study period (on the 29^th^ day),blood samples were collected from all the animals by retro-orbital plexuses puncture under light ether anesthesia with the help of capillary tube [11]. Blood was collected into sterile centrifuge tubes with K_3_ ethylenediaminetetraacetic acid (EDTA) as an anticoagulant for hematology and plain centrifuge tubes without anticoagulant for serum biochemistry. Blood was allowed to clot at room temperature (26±2°C) and serum was harvested after centrifugation at 3000 rpm for 10 min at 10°C (Eppendorf 5804 R, Germany) and stored at −35°C for analysis within 24 h. After collection of blood samples, all those rats were sacrificed humanely for checking gross and histopathological alterations in kidney. Tissue samples were collected and preserved in 10% formalin solution for histopathological examination.

### Estimation of hematological and serum biochemical parameters

Blood samples collected in test tubes with K_3_EDTA were utilized for estimation of various hematological parameters by hematology auto analyzer (Mindray BC-2800 Vet, China). On the day of blood collection, red blood cells, hemoglobin (Hb), packed cell volume mean corpuscular volume (MCV), mean corpuscular Hb (MCH) and MCH concentration (MCHC), total leucocytes counts white blood cells and platelets were estimated. Serum biochemical parameters were estimated in clinical serum biochemistry analyzer (NOVA 2021 Biochemistry Analyzer, Analytical Technologies Limited, Gujarat, India) including creatinine, uric acid and blood urea nitrogen (BUN). Serum XO activity was detected by using Coupled enzyme assay kit, purchased from Sigma-Aldrich (Spruce Street, St. Louis, MO, USA) by following the manufacturer’s protocol. XO activity was determined by spectrophotometric multiwell plate reader (Infinite M200, NanoQuant, TECAN) having 570 nm wavelength. XO activity was reported as milliunit/mL, where one milliunit of XO is defined as the amount of enzyme that catalyzes the oxidation of xanthine, yielding 1.0 µmol of uric acid and hydrogen peroxide per minute at 25°C.

### Histopathology

After opening the carcass, gross lesions were recorded and collected tissues were fixed in the formalin. The formalin fixed tissues were processed by paraffin wax embedding method of tissue sectioning. Sections from the kidney tissues were cut at 5-6 µ thickness with automatic section cutting machine (Leica, Automatic Microtome Machine, Germany) and were stained with hematoxylin and eosin stains.

### Statistical analysis

All the data have been presented as a mean±standard error. Statistical comparisons of the results were made using one-way analysis of variance using computer software SPSS (Version 20). Significant differences (p<0.05) between different experimental groups were analyzed by Duncan’s test.

## Results

Effect of repeated oral administration of aqueous and alcoholic extracts of *P. emblica* fruits for 28 days in normal and gouty rats on various hematological and serum biochemical parameters are presented in Tables-1 and -2, respectively. There was significant (p<0.05) increase in platelets counts in gout control rats as compared to vehicle control group. Animals of Group III, which was given standard treatment allopurinol showed significant reduction whereas animals of Groups IV, V, VI, and VII, which were given aqueous and alcoholic extracts of *P. emblica* revealed slight reduction in platelets counts as compared to gout control group. Other hematological parameters did not reveal any significant changes in any experimental groups.

**Table-1:** Hematological parameters in different experimental groups (n=6), (mean±SE).

Groups	RBC (10^6^/µL)	Hb (g/dL)	PCV (%)	MCV (fL)	MCH (pg)	MCHC (g/dL)	TLC (10^3^/µL)	PLT (10^5^/µL)
I	8.27±0.27	17.88±0.42	43.88±0.43	53.78±0.29	20.69±0.48	39.76±0.29	9.25±0.67	9.58±0.85^a^
II	8.23±0.25	17.28±0.41	42.35±0.28	53.27±0.66	21.35±0.49	40.55±0.84	8.01±0.58	12.92±0.43^c^
III	8.55±0.30	17.60±0.64	43.45±0.34	53.53±0.40	21.42±0.42	40.49±0.30	8.88±0.38	10.52±0.45^ab^
IV	8.97±0.33	18.70±0.35	44.51±0.50	52.50±0.55	20.50±1.20	40.33±0.45	8.28±0.23	11.79±0.34^bc^
V	9.05±0.44	18.26±0.57	44.23±0.39	52.85±0.71	21.80±0.90	39.97±0.42	9.33±0.37	10.81±0.96^abc^
VI	8.78±0.37	18.53±0.39	43.20±1.14	52.97±0.37	20.77±0.39	39.25±0.75	9.47±0.43	11.67±0.38^abc^
VII	9.01±0.67	18.80±0.66	43.61±0.38	53.60±0.54	21.51±0.51	40.90±0.38	9.81±0.80	11.88±0.93^bc^

Mean value with dissimilar superscript in a column vary significantly at p<0.05; Treatment groups: Groups II-VII given potassium oxonate (250 mg/kg) for induction of gout; I: Vehicle control; II: Gout control; III: Standard treatment control; IV: Aqueous Extract *P. emblica* (200 mg/kg); V: Aqueous extract *P. emblica* (400 mg/kg); VI: Alcoholic extract *P. emblica* (200 mg/kg); VII: Alcoholic Extract *P. emblica* (400 mg/kg). RBC=Red blood cells; Hb=Hemoglobin; PCV=Packed cell volume; MCV=Mean corpuscular volume; MCH=Mean corpuscular hemoglobin; MCHC=Mean corpuscular hemoglobin concentration; TLC=Total leukocyte counts, PLT=Platelets counts, SE=Standard error, *P. emblica=Phyllanthus emblica*

**Table-2:** Serum biochemical parameters in different experimental groups (n=6), (mean±SE).

Groups	Creatinine (mg/dL)	Uric acid (mg/dL)	BUN (mg/dL)	XO activity (milliunit/mL)	XO inhibition %
I	0.39±0.03^a^	8.86±0.14^a^	18.41±0.31^a^	1.69±0.17^a^	-
II	3.01±0.07^e^	15.50±0.37^d^	23.83±0.81^c^	3.33±0.51^b^	-
III	0.45±0.09^a^	10.06±0.28^b^	19.05±0.44^a^	1.70±0.30^a^	48.94
IV	1.51±0.17^c^	12.42±0.31^c^	21.02±0.29^b^	2.08±0.08^a^	37.53
V	1.05±0.04^b^	11.92±0.29^c^	20.85±0.49^b^	1.88±0.12^a^	43.54
VI	1.87±0.06^d^	12.79±0.21^c^	21.10±0.44^b^	1.85±0.34^a^	44.44
VII	1.79±0.10^d^	12.72±0.32^c^	20.90±0.35^b^	1.80±0.19^a^	45.94

Mean value with dissimilar superscript in a column vary significantly at p<0.05; Treatment groups: Groups II-VII given potassium oxonate (250 mg/kg) for induction of gout; I: Vehicle control; II: Gout control; III: Standard treatment control; IV: Aqueous extract *P. emblica* (200 mg/kg); V: Aqueous Extract *P. emblica* (400 mg/kg); VI: Alcoholic extract *P. emblica* (200 mg/kg); VII: Alcoholic extract *P. emblica* (400 mg/kg). BUN=Blood urea nitrogen, XO=Xanthine oxidase, SE=Standard error, *P. emblica=Phyllanthus emblica*

There was significant (p<0.05) increase in serum creatinine, uric acid, BUN, and XO enzyme level in the rats of gout control group as compared to vehicle control group whereas significant (p<0.05) decrease in the allopurinol treated group as compared to gout control rats. Animals of Groups IV, V, VI, and VII, which were given aqueous and alcoholic extracts of *P. emblica* also revealed significant (p<0.05) reduction in level of serum creatinine, uric acid, BUN, and XO enzyme level in dose-dependent manner as compared to gout control group.

**Figure-2:**
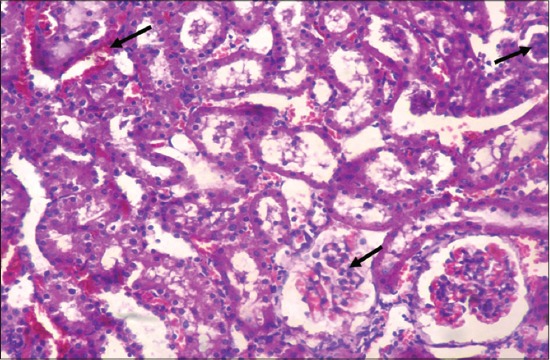
Section of kidney of rat from gout control group (Group II) showing atrophy of glomeruli, severe congestion and hemorrhage with degeneration and necrosis of renal tubular epithelium (H and E,×240).

**Figure-3:**
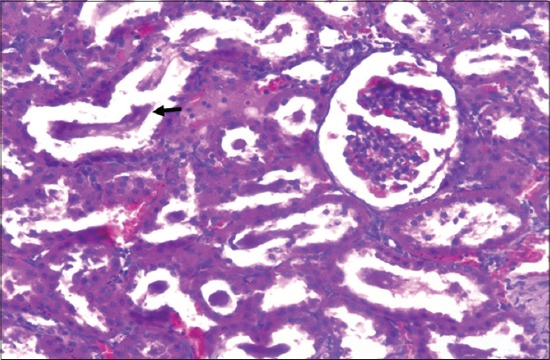
Section of kidney of rat from gout control group (Group II) showing presence of proteinaceous cast in renal tubular lumen (H and E,×240).

**Figure-4:**
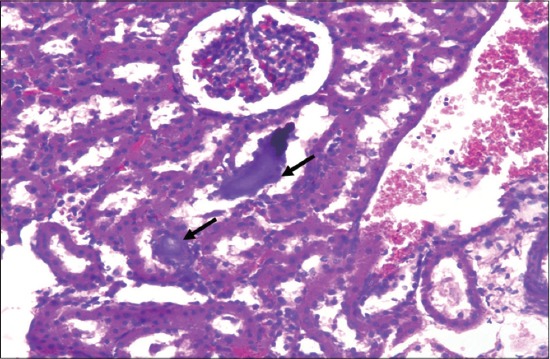
Section of kidney from gout control rat (Group II) showing deposition of urate crystals in renal parenchyma (H and E,×240).

Detailed post-mortem examination of all the animals of different groups was performed on the 29^th^ day. Kidneys collected from rats of gout control group showed paleness as compared to kidneys of vehicle control group. Histopathological changes in kidneys of rats of different groups revealed dose dependent varying degree of degenerative, as well as necrotic changes. The lesions were mild to severe in nature, and the extent of kidney damage was directly correlated to the degree of urate deposition. Microscopic changes in kidneys of animals from Group II revealed atrophy of glomeruli and desquamation of tubular epithelium along with severe congestion, hemorrhage, degeneration, and necrosis of renal tubular epithelium (Figure-2). Sections of kidney from Group II showed the presence of proteinaceous cast in renal tubular lumen (Figure-3). Further, these lesions were manifested by deposition of urate crystals in renal parenchyma (Figure-4).

**Figure-5:**
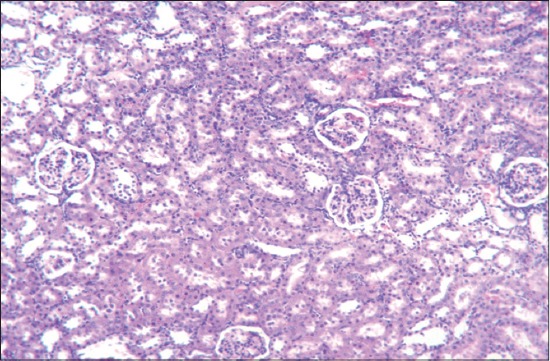
Section of kidney from rat of Group VII treated with alcoholic extract of *Phyllanthus emblica* at 400 mg/kg revealing significant improvements in renal histomorphological structures (H and E,×120).

Simultaneous treatment of hyperuricemic rats with aqueous and alcoholic extracts of *P. emblica* at 200 and 400 mg/kg body weight and standard treatment allopurinol at 5 mg/kg body weight revealed significant improvements in renal histomorphological structures (Figure-5), indicating ameliorating potential of *P. emblica* on gout lesions in hyperuricemic rats. All the organs collected from vehicle control rats did not show any macroscopic and microscopic alterations.

## Discussion

Antigout activity of *P. emblica* fruits in PO induced gout in rats was investigated in the present study. Intra-peritoneal injections of PO at 250 mg/kg body weight (daily for 28 days) has lead to increased serum concentration of creatinine, uric acid, BUN, and XO enzyme level(as indicator of impaired kidney function) along with deposition of urate crystals in renal parenchyma, atrophy of glomeruli and desquamation of tubular epithelium, severe congestion and hemorrhage with degeneration and necrosis of renal tubular epithelium and presence of proteinaceous cast in renal tubular lumen in rats of gout control group. These observations suggest that chronic administration of PO develops gout lesions. The histopathological observations were consistent with these biochemical changes. Such changes in serum biochemical parameters established successful gout rat model. Similar to these findings, plasma samples collected from rats after 3 and 6 h from PO injection at 250 mg/kg body weight have also demonstrated rise in uric acid [12]. Changes in serum biochemical parameters of gout control group in present study also agree with the work of Haidari *et al*. [13],where intra-peritoneal injection of PO at 250 mg/kg body weight was shown to cause significant increase in uric acid and XO level as compared to the vehicle control rats. These biochemical and histopathological findings have also been previously reported by Sanchez-Lozada *et al*. [14] and support the direct involvement of hyperuricemia in induction of gout and other renal disease.

In the present study, there was significant (p<0.05) increase in mean platelets counts in rats of gout control group as compared to vehicle control rats. This might be occurred due to inflammation and it is also a representation of chronic inflammatory condition [15]. Increased number of platelets counts is also observed in other inflammatory conditions like rheumatoid arthritis [16,17], inflammatory bowel disease [18] and nephritis. On activation, platelets secrete a large number of biologically active molecules, which are able to induce or amplify an inflammatory process. Whereas gouty rats treated with aqueous and alcoholic extracts of *P. emblica* fruits at 200 and 400 mg/kg body weight showed non-significant reduction in mean platelets counts as compared to rats of gout control group, however the platelets counts did not reach to the normal level in these animals. The same type of study was reported by Savala *et al*. [19] who studied the effect of *Emblica officinalis* formulation on hematological parameters in wistar rats and the results showed that there was a better platelets counts of the treated rats with respect to control rats.

Present study indicated that the group of rats given standard reference compound allopurinol at 5 mg/kg body weight significantly (p<0.01) reduced serum creatinine, uric acid, BUN, and XO enzyme level in hyperuricemic rats. Here, the extent of reduction in XO enzyme level by allopurinol was much higher than that observed with *P. emblica* extracts in hyperuricemic rats. Hyperuricemic rats receiving aqueous and alcoholic extracts of *P. emblica* at 200 and 400 mg/kg body weight also showed significant reduction in serum creatinine, uric acid, BUN, and XO enzyme level as compared to rats of gout control group in dose- dependent manner.

Similarly, significant decline in elevated uric acid has also been reported by Quinto *et al*. [20] who studied usefulness of fruit of *Morinda citrifolia* (Noni) in the treatment of gout where PO induced gout in Sprague-Dawley rats were used to study the hypouricemic effects through measurement of uric acid levels in both acute and sub-acute oral settings. Acute exploratory screening showed that significant hypouricemia was achieved with the lyophilized fruit juice at 4.1 g/kg, and with the lyophilized commercial noni juice at 10 g/kg, with both doses given orally. Similar results were also observed by Basah *et al*. [21] who studied the effect of *Justicia gendarussa* leaves extract on oxonate-induced hyperuricemic male albino rats. Three group doses variations of *J. gendarussa* extract like 1.3 g/kg, 2.6 g/kg and 5.2 g/kg body weight showed dose dependent reduction in uric acid level of rats. Similar observations were also noticed by Hu *et al*. [22] who reported the potent anti-hyperuricemic activities of *Fructus gardenia* extract in mice. Serum and urinary levels of BUN and fractional excretion of uric acid were measured where BUN and urate levels were significantly reduced in hyperuricemic mice treated with *Fructus gardenia* extract.

Numerous studies have showed that phenolic compounds found in plants, such as anthocyanins and quercetin, which are structurally related to xanthine, inhibit XO activity, thus reducing hyperuricemia [23]. However, there is no parallel relationship between the extent of the hypouricemic action and the reduction in the enzyme activity.

Similarly, Haidari *et al*. [13] also reported the effect of tart cherry juice on hepatic xanthine oxidoreductase activity in normal and hyperuricemic rats where tart cherry juice treatment inhibited hepatic XO activity. Finding of present study agrees with the results of Raju *et al*. [24] who studied the *in vivo* hypouricemic activity of the various fractions of the hydromethanolic extract of the leaves of *Erythrina stricta* roxb (papilionacea) using oxonate-induced hyperuricemic mice. The pet-ether, chloroform and ethyl acetate fractions when administered to hyperuricemic mice produced significant inhibitory actions on the XO enzyme.

The fruit of *P. emblica* is known as a richest source of ascorbic acid [25]. In addition to this, several active tannoid principles (emblicanin A, emblicanin B, punigluconin,and pedunculagin) and other polyphenols: Flavonoids, kaempferol, ellagic acid, and gallic acid have been identified for their health benefits [26]. The renoprotective effect of gallic acid from antioxidant and anti-inflammatory properties was demonstrated in many experimental studies in acute kidney injury against lindane [27], sodium fluoride [28] and chronic kidney disease [29].

The results also support the hypothesis that vitamin C present in *P. emblica* might be an effective dietary approach in the prevention and management of gout and its related diseases [30]. Previously, it has been reported that vitamin C can lower serum uric acid via a direct uricosuric effect, which is due to a competition for renal reabsorption of uric acid via an anion-exchange transport system at the proximal tubule or by increasing glomerular filtration rate and thus providing another potential mechanism for the uricosuric effect of vitamin C intake.

## Conclusion

Our study shows that 28 days repeated oral administration of aqueous and alcoholic extracts of *P. emblica* fruits at 200 and 400 mg/kg body weight produce antigout effect in rats in dose dependent manner. However, based on the results obtained in this study, it seems that more detail studies on the mechanisms of antigout activity of this plant and its probable use in gout sufferers are still to be investigated. Finally, we suggest that *P. emblica* has potential for newer therapeutic applications in the future.

## Authors’ Contributions

This study is the major component of the work toward the M. V. Sc. thesis of the first author VNS. AMT and KAS provided guidance during the entire experiment and corrected manuscript. ACP provided SPSS software and helped in data analysis. PGP and ASP helped in blood collection from rats, histopathological examination and biochemical analysis. All authors have read and approved the final version of the manuscript.
